# Site directed mutagenesis studies on HIV-1 reverse transcriptase (RT) shed light on the mechanism of action of a new Ribonuclease H/ DNA polymerase RT dual inhibitor

**DOI:** 10.1186/1742-4690-10-S1-P19

**Published:** 2013-09-19

**Authors:** Angela Corona, Rita Meleddu, Francesca Esposito, Simona Distinto, Giulia Bianco, Elias Maccioni, Stuart SF Le Grice, Enzo Tramontano

**Affiliations:** 1 Department of Life and Environmental Sciences, University of Cagliari, Cagliari, ltaly; 2 National Cancer Institute, Frederick, MD, USA

## Background

The viral reverse transcriptase (RT) is a major target in the therapeutic strategy against the human immunodeficiency virus (HIV). This multifunctional enzyme performs the viral genome replication by two associated functions: DNA polymerase and ribonuclease H (RNase H). Both activities are essential for viral replication and validated targets. Currently, none of the approved antiviral drugs acts on both activities, although few classes of compounds have been reported to inhibit both RT functions *in vitro *[1]. In a previous virtual screening [2] we identified a new isatine-based scaffold for dual functions RT inhibitors that was further derivatized.

## Materials and methods

We performed blind docking studies on wild type HIV-1 RT with the isatine-derivative RMNC6. We employed the QM-polarized ligand docking protocol utilizing Glide version 4.5, qsite version 4.5, jaguar 7.0 and maestro 8.5 (Schrodinger Inc, Portland, USA). Residues hypothesized to be critical for compound binding were mutated by site-directed mutagenesis. Mutants RTs were tested for susceptibility to RMNC6 on both RNase H and RNA dependent DNA polymerase activities.

## Results

The isatine-derivative RMNC6 inhibited the RT-associated RNase H and DNA polymerase activities with IC_50_ values of 1.4 and 9.8 µM concentration, respectively. Blind docking analysis suggested that RMNC6 could bind to two different RT pockets: the first one close to the RNase H active site, the second one near to the polymerase active site. Conserved residues close to the primer grip regions were individuated as potentially critical for inhibitor binding and mutated. Mutagenesis results identified few amino acid residues in the RNase H domain that are essential for RNase H inhibition by RMNC6 but do not influence its DNA polymerase inhibition. By contrast, mutation of some amino acid residues in the DNA polymerase domain affects up to 5 fold the inhibition of both DNA polymerase and RNase H by RMNC6.

## Conclusions

The new isatine-derivative RMNC6 was shown to inhibit both RT functions in the low micromolar range. Molecular modeling and site-directed mutagenesis studies supported the hypothesis that RMNC6 could bind to two different RT sites. Binding to the RNase H site appears to be responsible for RNase H inhibition, while binding to the DNA polymerase site seems to affect both RT-associated functions by both short- and long-range effects.
